# A pilot study of healing critical-sized calvarial defects by LL-37-generated monoosteophils

**DOI:** 10.3389/fbioe.2025.1583496

**Published:** 2025-07-14

**Authors:** Keith Le, Huinan Liu, Chaoxing Zhang, Zhuo Li, Tove Olafsen, Yuman Fong, John E. Shively, Zhifang Zhang

**Affiliations:** ^1^ Department of Immunology and Theranostics, Arthur Riggs Diabetes and Metabolism Research Institute of City of Hope National Medical Center, Duarte, CA, United States; ^2^ Department of Bioengineering, University of California Riverside, Riverside, CA, United States; ^3^ Electron Microscopy Core Facility, City of Hope National Medical Center, Duarte, CA, United States; ^4^ Small Animal Imaging Core, City of Hope National Medical Center, Duarte, CA, United States; ^5^ Department of Surgery, City of Hope National Medical Center, Duarte, CA, United States

**Keywords:** critical-size calvarial defect, mineralization, bone nodule formation, bone repair, monoosteophil

## Abstract

**Introduction:**

Monoosteophils, derived from LL-37-treated monocytes, are a novel type of calcifying/bone forming cells. We have shown that monoosteophils can form bone-like nodules *in vitro* and accelerate bone repair in a drilled femur defect model. Here, we explored the bone repair function of monoosteophils in a mouse model of critical-sized calvarial defect and the mechanism of bone nodule formation of monoosteophils *in vitro*.

**Methods:**

Human monocytes were isolated from peripheral blood and differentiated into monoosteophils. Critical-sized (5 mm-diameter) calvarial defects in the parietal bone of adult male NOD/SCID mice were implanted with either 1-day untreated human monocytes, 1-day LL-37 treated human monocytes (monoosteophils), 1-day human monocytes plus hydroxyapatite nanoparticles or 1-day human monoosteophils plus hydroxyapatite nanoparticles. Micro-computed tomography (µCT) was used for assessment of bone formation in the mouse model. Alizarin Red S staining (ARS), FAM-alendronate staining, light and fluorescence microscopy, scanning electron microscopy (SEM), energy dispersive X-ray spectroscopy (EDS) and transmitted electron microscopy (TEM) were used to examine bone nodule formation *in vitro*.

**Results:**

The most complete healing (80%) was observed for monoosteophils plus nano-scale hydroxyapatite. The results of a dose response study (5 × 10^6^, 2.5 × 10^6^, 1.25 × 10^6^ and 0.625 × 10^6^ MOP cells) showed that monoosteophil cell counts as low as 0.625 × 10^6^ cells were able to significantly repair the defect area over a short-term observation period of 4 weeks. Mechanistic *in vitro* studies using ARS and FAM-alendronate staining showed that monoosteophils form bone nodule in αMEM medium supplemented with 2.5 mM CaCl_2_. SEM/EDS analysis confirmed that the bone nodules consisted of phosphorus, calcium, oxygen, and sodium. Monoosteophils in culturing condition formed the unique granules in the cytoplasm consisting of phosphorus, calcium, oxygen, and sodium evidenced by SEM/EDS.

**Discussion:**

We now demonstrate that the bone repair function of monoosteophils requires hydroxyapatite through intracellular nodule formation and monoosteophils are capable of filling in large calvarial defects in our pilot study. These observations may have important implications in facilitating the development of therapeutic applications for clinically challenging bone repairs and the understanding of pathological mineralization.

## 1 Introduction

Bone fracture is a very frequent cause of morbidity with one third of males and one half of females affected, at least once in their lifetime (Papachristou et al., 2021). Two to eleven percent of fractures, depending on which long bone has been traumatized, fail to unite, resulting in non-unions (Papachristou et al., 2021). Various types of bone defects are major challenges facing clinical practice (Agarwal and Garcia, 2015). The socioeconomic burden imposed on the healthcare system by fractures has pushed the scientific community towards a synchronized effort to develop more effective therapeutic interventions for the successful management of bone injuries (Melton et al., 2003).

For cell therapy of bone defects, osteoblasts are considered the ideal cellular component of engineered bone implants. However, technical difficulties, such as harvesting and expanding osteoblasts into meaningful numbers, undermine the benefits of using primary cells. Consequently, various types of stem cells have been largely proposed as a viable and easy source of osteoblast progenitors for the generation of engineered bone implants. Mesenchymal stem cells (MSCs) are multipotent stem cells that differentiate into many different types of tissues, including bone (osteoblasts), cartilage (chondrocytes), muscle (myocytes), and fat (adipocytes). Adult MSCs, isolated from many different tissues including bone marrow, skeletal muscle, synovial membrane, and adipose tissue, act as an inducible reserve force for tissue regeneration after injury (Caplan and Correa, 2011), and therefore have been studied extensively for their therapeutic potential in fracture healing and bone regeneration. Although MSCs have been in clinical trials for over two decades, their use remains controversial, due to the lack of published clinical trials, controversies in the published results, and variability in the methods of trans-differentiation and treatment strategies (Oryan et al., 2017).

Monocytes, a source of adult stem cells (Ungefroren et al., 2016; Seta and Kuwana, 2010), are a readily available alternative source of cells that can be potentially differentiated in bone repair cells. In fact, monocytes treated with the natural anti-microbial peptide LL-37 form a novel type of calcifying/bone forming cell we have termed monoosteophil (Zhang and Shively, 2010; Zhang and Shively, 2013; Zhang et al., 2020). Monoosteophils are characterized by the phenotypic analysis as CD45^+^α3^+^α3β1^+^CD34^−^CD14^−^ bone alkaline phosphatase negative cells, expressing characteristic markers of both osteoblasts and osteoclasts. We have shown that monoosteophils have the ability to form bone-like nodules *in vitro*, ectopic bone *in vivo* and accelerate bone repair in a drilled femur defect model (Zhang and Shively, 2010; Zhang and Shively, 2013). Here, a critical-sized calvarial defect model was used to further investigate the bone repair function of monoosteophils, along with the *in vitro* mechanism of bone nodule formation.

## 2 Materials and methods

### 2.1 Reagents

LL-37 was synthesized by standard FMOC chemistry as previous described (Zhang and Shively, 2010). EasySep^®^ Human Monocyte Enrichment Kit (catalog# 19059) were purchased from StemCell Technologies Inc. (Vancouver, Canada). Recombinant human M-CSF (catalog# CYT-308) and RANKL (catalogy# CYT-334) from ProSpec Tany TechnoGene Ltd. (Rehovot 7670308, Israel). MEM alpha (αMEM, Catalog# 12571-063) was purchased from Gibco (Montana, USA). Bio-Gel HT hydroxyapatite (catalog# 1300151), CHT ceramic hydroxyapatite (catalog# 1572000, 20 µm particle size and catalog#1584000, 40 µm particle size) were purchased from BIO-RAD (Hercules, CA 94547, USA). Hydroxyapatite nanoparticles (Nano-HA, particle diameter, 63 ± 50 nm) were produced by Dr. Huinan Liu at the University of California, Riverside (Johnson et al., 2013). FITC anti-human CD14 Antibody (catalog# 325604, clone: HCD14) and cell staining buffer (catalog# 420201) were purchased from Biolegend (San Diego, CA, USA). RPMI1640 medium (catalog# 10-040-CM), antibiotic antimycotic solution (catalog# 30-004-Cl), 0.05% trypsin (catalog# 25-051-C1) and PBS (catalog# 21-040-CV) were purchased from Mediatech Inc. (Corning brand, Manassas, VA, USA). Fetal Bovine Serum (catalog# FB-12) was purchased from Omega Scientific Inc. (Tarzana, CA, USA). Collagen sponge for 35 mm culture dish was purchased from KOKEN Co. Ltd. (catalog # CS-35, Tokyo, Japan). Corning^®^ Matrigel^®^ Basement Membrane Matrix was purchased from Millipore-Sigma (catalog# CLS356237, St. Louis, MO). Alizarin Red S staining kit (catalog# 8678) was purchased from ScienCell Research Laboratories (Carlsbad, CA 92008, USA). Ficoll-Paque Plus (catalog# GE17-1440-03) density gradient was purchased from GE Healthcare Biosciences (Pittsburgh, PA, USA). Paraformaldehyde (PFA) 32% solution was purchased from Electron Microscopy Sciences (catalog# 15714, Hatfield, PA19440) and diluted to 4% PFA by using 1x PBS. Corning^®^ BioCoat^®^ Collagen I-coated Plates (catalog#354408 for 24-well and catalog#354505 for 48-well plates) were purchased from VWR (West Chester, PA, USA). Carboxyfluorescein-alendronate (FAM-alendronate) was synthesized as previously described (Ahrens et al., 2017).

### 2.2 Monocyte isolation and cell differentiation

The use of anonymous discard blood samples without the requirement for informed consents was approved by the City of Hope Institutional Review Board (IRB # 99132) (Zhang and Shively, 2010). Peripheral blood mononuclear cells (PBMCs) were isolated from citrated human blood (discard blood from anonymous donors) by centrifugation over Ficoll-Paque Plus density gradient. Monocytes were separated from PBMCs using EasySep^®^ Human Monocyte Enrichment Kit. The purified monocytes were stained with anti-CD14-FITC and analyzed using flow cytometry on a FACSCanton II. Monocytes with >95% purity were suspended at 1 × 10^6^ cells/mL in RPMI 1640 medium or αMEM supplemented with 10% FBS and 1% antibiotic antimycotic solution. For cell differentiation, monocytes were treated with medium only (control), 5 µM LL-37 (monoosteophils) or RANKL/M-CSF (both at 25 ng/mL, osteoclasts). Cell morphology was imaged by using Leica DMI 3000B inverted microscope (Leica Microsystems Inc., Bannockburn, IL60015).

### 2.3 Alizarin red sulfonate (ARS) and FAM-alendronate staining

Calcium deposits in cell culture plate were evaluated by using ARS and FAM-alendronate staining (Ahrens et al., 2017; Li et al., 2020). For ARS staining, cells in the 24-well plate were washed twice with PBS, fixed with 4% paraformaldehyde (PFA) for 15min, washed in distilled water three times at 5 min interval. Then cells were incubated with ARS staining solution at room temperature for 30 min. Excess staining was washed three times in distilled water to remove the unbound dye. The morphological alterations were examined and photographed using Leica DMI 3000B inverted microscope (Leica Microsystems Inc., Bannockburn, IL 60015, USA). For FAM-alendronate staining, cells in the 24-well plate were washed twice with PBS, fixed with 4% PFA, washed in PBS, stained with FAM-alendronate for 30 min, washed with PBS, and imaged using Leica DMI 3000B inverted microscope (Zhang and Shively, 2010).

### 2.4 Scanning electron microscopy (SEM) and energy dispersive X-ray spectroscopy (EDS) analysis

Monocytes at the cell concentration of 1 × 10^6^ cells/mL in 10% FBS αMEM were incubated at 5% CO_2_ atmosphere with 5 µM LL-37 or M-CSF/RANKL (both at 25 μg/mL) for 6 days. Then cells in the plates were incubated with 10% FBS αMEM supplemented with 2.5 mM CaCl_2_ for 3 weeks. For SEM, cells in the wells of plate were fixed with 2% glutaraldehyde in 0.1M Cacodylate buffer (Na(CH3)2AsO2 ·3H2O), pH7.2. The cells were washed three times with 0.1M Cacodylate buffer, pH7.2, post-fixed with 1% OsO4 in 0.1M Cacodylate buffer for 30 min and washed three times with 0.1M Cacodylate buffer. The samples were then dehydrated through 30%, 50%, 60%, 70%, 80%, 95% ethanol, 100% absolute ethanol (twice). The samples were dried in a critical point dryer. The dried samples were then coated with gold and palladium (Au: Pd 60/40 ratio) in a Cressington 308R coating system. For TEM to view intracellular nodule formation of monoosteophils, the cells were harvested with 0.05% trypsin, washed with PBS, and fixed with 2% glutaraldehyde in 0.1M Cacodylate buffer (Na(CH3)2AsO2 ·3H2O), pH7.2, at 4°C, overnight. The cell pellets were washed three times with 0.1M Cacodylate buffer, pH7.2, post-fixed with 1% OsO4 in 0.1M Cacodylate buffer for 30 min and washed three times with 0.1M Cacodylate buffer. The samples were dehydrated, infiltrated, and embedded in Eponate. Thin sections (∼100 nm thick) were cut using a Leica Ultra cut UCT ultramicrotome with a diamond knife and deposited on a silicon chip substrate (SPI Supplies Item # 4136SC-AB). For EDS, both the coated samples and deposited thin sections were imaged with a Zeiss Sigma VP field-emission scanning electron microscope equipped with an Oxford X-max x-ray detector for identification of the elemental composition of monoosteophil-formed nodules. The accelerating voltage was 10–20 keV. Quantification of granule element intensity is analyzed using Image Pro Premier (Media Cybernetics).

### 2.5 Mouse model of critical-sized calvarial defect

General anesthesia was performed by intraperitoneal injection with ketamine hydrochloride (120 mg/kg) and xylazine hydrochloride (10 mg/kg) in male NOD/SCID mice. Nonhealing, critical-sized calvarial defects (5 mm-diameter, 19.6 mm^2^) were created in the right or left parietal bone using a dental drill (Yang et al., 2012). Briefly, under sterile conditions, a sagittal incision was made from the orbital ridge to 5 mm behind the ears. Skin flaps on either side were retracted to expose the calvaria. A MICRO-DRILL SYSTEM (120 vac, catalog# 110-4102, Circuit Medic, Haverhill, MA 01835, USA) and Dremel handheld drill (Racine, WI) fitted with a stainless-steel trephine drill bit was used at low speed to create a full-thickness 5 mm-diameter calvarial defect (19.6 mm^2^) on the right or left parietal bone of each mouse. Sterile phosphate buffered saline (PBS) with 1000 IU/mL penicillin and 1 mg/mL streptomycin (35 mL/kg) was used for irrigation during drilling. For defects considered acute, a collagen scaffold with cells was placed immediately at the time of injury. In preparation for cell engraftment, scaffolds (collagen sponge) were seeded with human 1-day-old monocytes (monocytes treated with PBS for 1 day, control) or 1-day-old monoosteophils (monocytes treated with 5 µM LL-37 for 1 day, MOP) or/and plus hydroxyapatite in a total volume of 25 µL PBS containing 50% Matrigel. The experiments were conducted in three conditions: group 1: 1-day monocyte control alone (3 × 10^6^ cells, n = 3) and 1-day monoosteophils alone (3 × 10^6^ cells, n = 3) imaged by using µCT on week 14 post-operation; group 2: 1-day monocytes with Nano-HA (Control + HA, 3 × 10^6^ cells, n = 3) and 1-day monoosteophils with Nano-HA (MOP + HA, 3 × 10^6^ cells, n = 6) imaged by using µCT over time (week 1, week 4, week 8 and week 13) post-operation; group 3: titration of 1-day monoosteophil numbers (MOP, 5 × 10^6^ cells, 2.5 × 10^6^ cells, 1.25 3 × 10^6^ cells and 0.625 × 10^6^ cells) with Nano-HA over a short-term observation period of 4 weeks (week 1, week 2 and week 4) post-operation. Finally, the skin was sutured with 4-0 vicryl and the animal was monitored per established postoperative animal care protocols. Pain medication Buprenorphine was subcutaneously injected before operation. All procedures were done in accordance with an animal protocol approved by the City of Hope National Medical Center Institutional Animal Care and Use Committee (IACUC# 09028).

### 2.6 MicroCT imaging and data analysis

To monitor the progression of bone repair, mice underwent serial µCT imaging at time points shown in the figure postoperatively. During scanning, the mice were sedated using the same isoflurane delivery procedure described previously (Zhang et al., 2022). Mice were examined using Siemens MicroCAT II Ultra Hi-Res (Siemens Medical Solutions, Knoxville, TN). Reconstruction of the calvarial defect region was performed following correction of the rotation center and calibration of the mineral density. Bone analysis was performed using MicroDicom software (Zhang and Shively, 2013).

### 2.7 Statistical analysis

Assay results are expressed as means ± SEM and unpaired Student’s t-tests were used for comparisons. All p-values are two sided. Data were analyzed with GraphPad Prism software (version 7.0, GraphPad Software, San Diego, CA, USA).

## 3 Results

### 3.1 Human monoosteophils partially heal critical-sized calvarial defects in NOD/SCID mice

**FIGURE 1:**
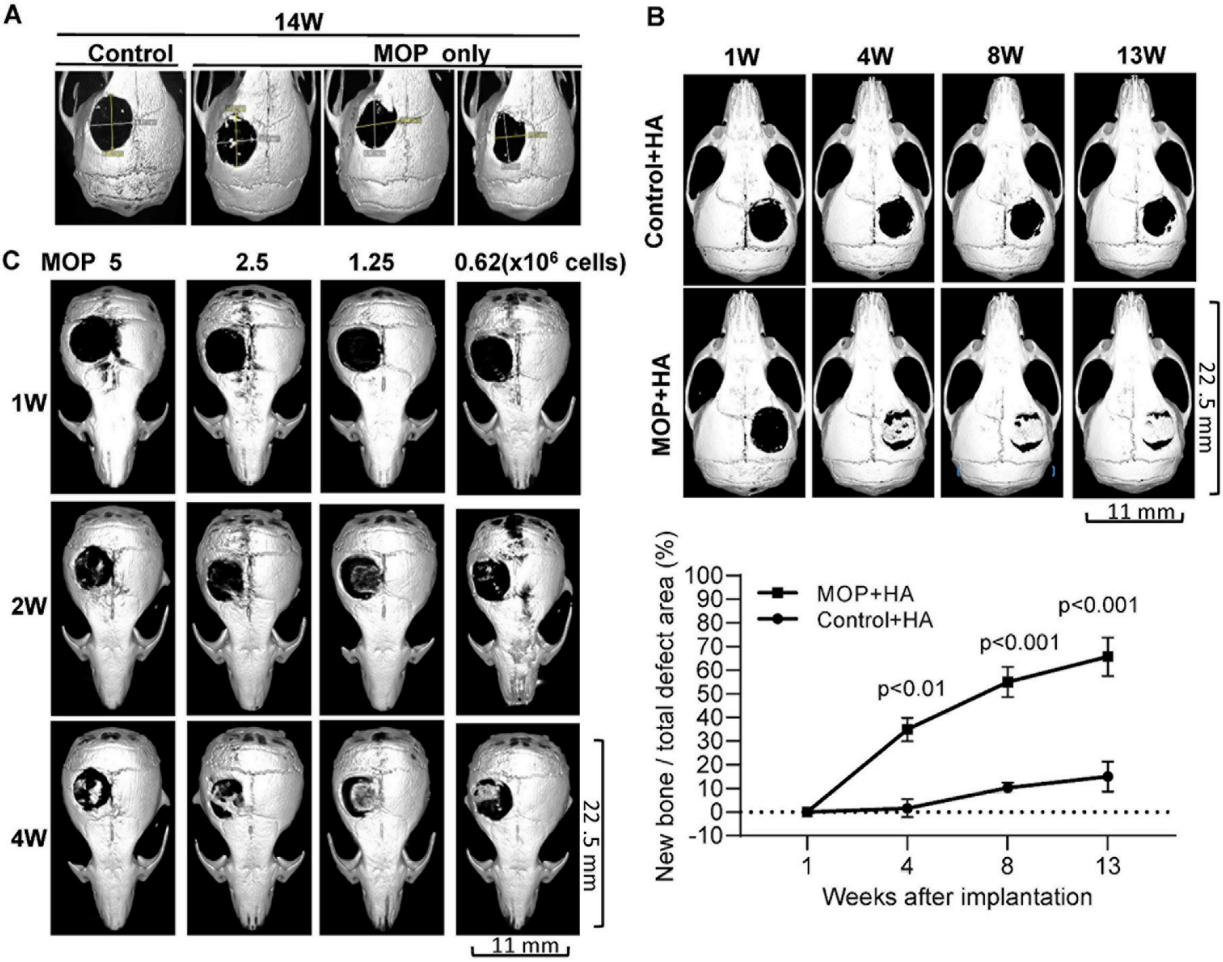
Calvarial healing of monoosteophils supplemented with hydroxyapatite in NOD/SCID mouse model. Critical-sized calvarial defects (5 mm) were created in the parietal bone of NOD/SCID mice. **(A)** Treatment groups included 1-day human monocyte control (Control, 3 × 10^6^ cells, *n* = 3, one representative shown) and 1-day human monoosteophils (MOP, 3 × 10^6^ cells, *n* = 3) with collagen sponge as scaffold and µCT images were acquired on week 14 after cells implanted. **(B)** Treatment groups included 1-day human monocyte control with hydroxyapatite (3 × 10^6^, Control + HA, *n* = 3) and 1-day human MOP with hydroxyapatite (MOP + HA, *n* = 6). One representative and statistical analysis of µCT imaging each group were shown over time. **(C)** Treatment groups included number titration of 1-day monoosteophils shown in the figure (MOP 5 × 10^6^ cells (*n* = 4), 2.5 × 10^6^ cells (*n* = 4), 1.25 × 10^6^ cells (*n* = 2), and 0.625 × 10^6^ cells (*n* = 4)) with hydroxyapatite in short-term observation. One representative of µCT imaging each group was shown over time. W, week.

To evaluate the potential of human monoosteophils for bone regeneration *in vivo*, 5-mm diameter bone defects (19.6 mm^2^) were created in the calvaria of NOD/SCID mice. The critical-sized calvarial defect model was chosen since these defects do not heal spontaneously (Lee et al., 2001; Krebsbach et al., 1998). The dynamic process of osteogenesis at the repair sites of the calvarial defect in mouse model was evaluated using µCT imaging over time postoperatively. As shown in Figure 1A, the control group of 1-day-old monocytes (control, 3 × 10^6^ cells) and 1-day-old monoosteophils (MOP, 3 × 10^6^ cells) on a collagen I sponge showed no healing or minor healing in the defect area on week 14 post operation. In Figure 1B, treatment with 1-day-old monocyte control with Nano-HA (Control + HA, 3 × 10^6^ cells) on a collagen I sponge showed minor healing at the edges but little evidence into the center of the defect area over 13 weeks. However, the 1-day-old monoosteophil treatment group on a collagen I sponge supplemented with Nano-HA (MOP + HA, 3 × 10^6^ cells) showed significant healing over the entire defect area. Quantification of µCT images was evaluated according to percent area healing of the defects (all images shown as Supplementary Figure 1). The maximum repair area is around 80% of defect areas with statistical significance (p < 0.01, or p < 0.001). From 7 weeks to 13 weeks, we observed active remodeling, characterized by the removal of excess structures in the defect area (Supplementary Figure 2A). One example of adventitious bone formation was found in an experimental animal in which the scaffold was displaced by wound scratching (Supplementary Figure 2B **)**.

A dose response study (5 × 10^6^, 2.5 × 10^6^, 1.25 × 10^6^ and 0.625 × 10^6^ MOP cells) was performed to determine the minimum number of monoosteophils with nano-sized hydroxyapatite required to repair a 5-mm diameter calvarial defect over a short-term observation period of 4 weeks. The results showed that monoosteophil cell counts as low as 0.625 × 10^6^ cells were able to significantly repair the defect area (Figure 1C). Similar to the MOP + HA treatment group (3 × 10^6^ MOPs) in shown in Supplementary Figure 2B, a lower dose of monoosteophils (1.25 × 10^6^) also showed bone formation process on intact calvaria implanted underneath the skin (Supplementary Figure 2C).

### 3.2 Formation of bone nodules by monoosteophils *in vitro*

**FIGURE 2:**
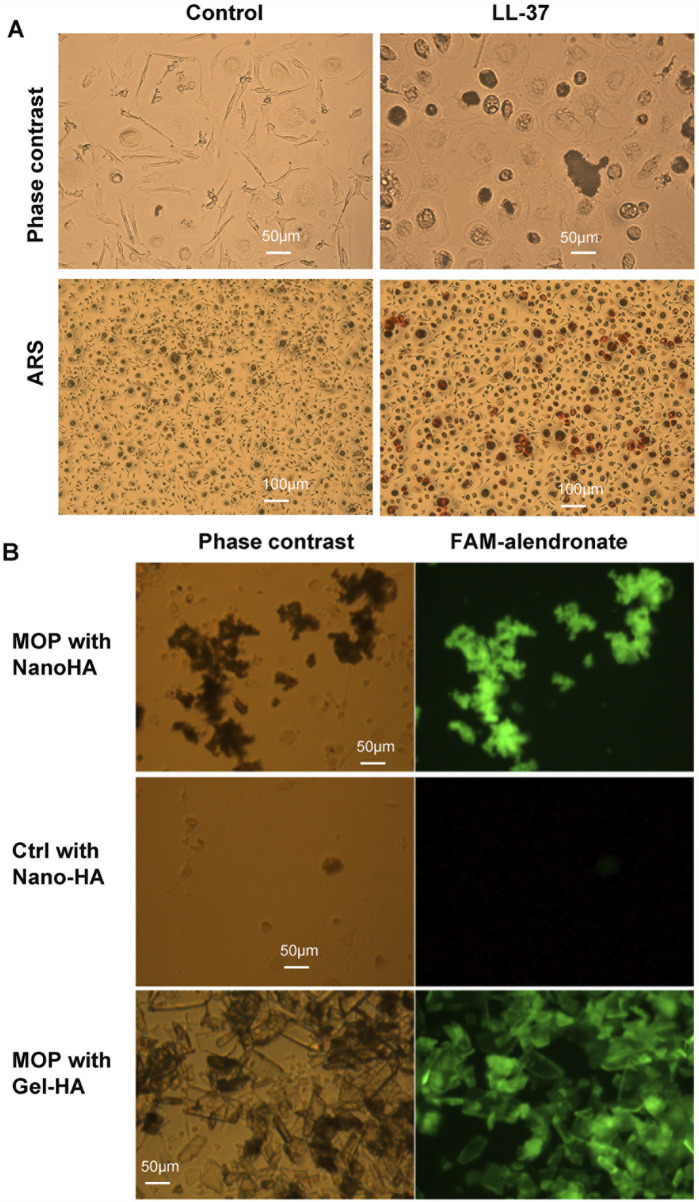
Alizarin Red S and FAM-alendronate staining of monoosteophil-formed nodules *in vitro*. **(A)** Monocytes were treated with 5 µM LL-37 or PBS control for 3 weeks in αMEM with 2.5 mM CaCl_2_. Then, cells in the plate were imaged before and after Alizarin Red S staining. Upper: ×20; Lower: ×10; ARS: Alizarin Red S staining. **(B)** Monocytes were treated with 5 µM LL-37 or PBS in the presence of nanoparticles of hydroxyapatite (Nano-HA) or gel hydroxyapatite (Gel-HA) for 10 weeks. Cells with built-structures were stained with FAM-alendronate and imaged with fluorescence microscopy.

To explore the mechanism of bone nodule formation by monoosteophils, monocytes were treated with PBS or 5 µM LL-37 in 10% FBS αMEM medium supplemented with 2.5 mM CaCl_2_ in collagen coated plates for 3 weeks and imaged before and after ARS staining (Figure 2A). Monocytes treated with LL-37, but not PBS control, built extracellular nodules seen by phase contrast microscopy that stained positive by ARS. In another culturing condition, monocytes in regular 10% FBS αMEM medium treated for 10 weeks with LL-37 plus Nano-HA, but not PBS control plus Nano-HA, also built extracellular bone nodules that stained with FAM-alendronate (Figure 2B), which serves as a second line of evidence for bone nodule formation (Li et al., 2020). Large size gel-hydroxyapatite cocultured with monocytes plus LL-37 for 10 weeks was used as positive staining control of FAM-alendronate and it is noted that dark color parts on the phase contrast colocalize with the bright part on the FAM-alendronate, which suggest the formed nodules by monoosteophils (Li et al., 2020). Thus, supplementation with CaCl_2_ or hydroxyapatite, as the source of both phosphorus and calcium, was required for monoosteophil nodule formation.

### 3.3 SEM/EDS analysis of monoosteophil-formed nodules *in vitro*

**FIGURE 3:**
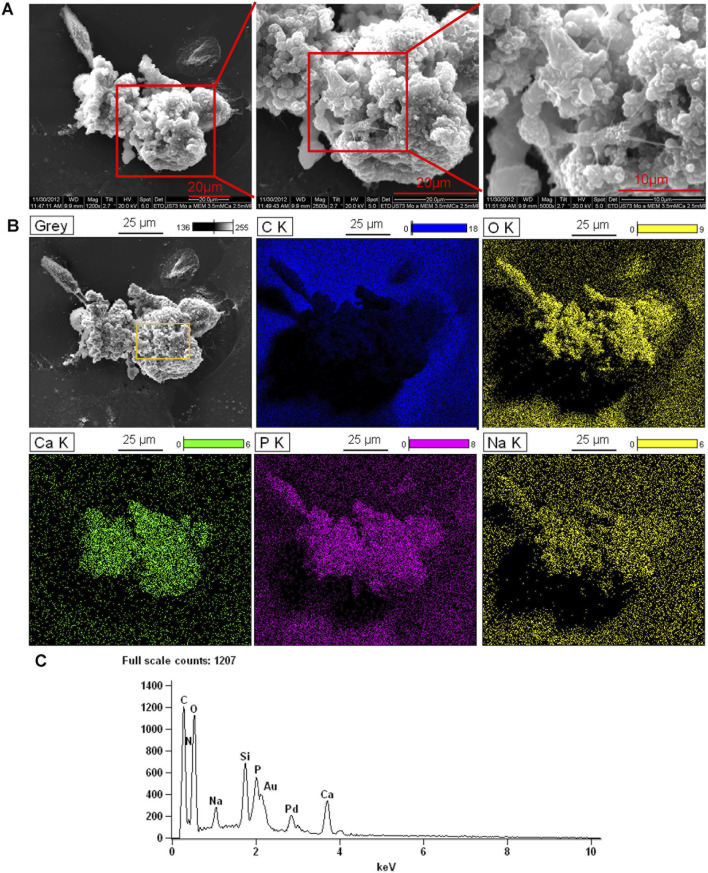
Bone formation of monoosteophils in αMEM media with 2.5 mM CaCl_2_. Monocytes were treated with LL-37 for 3 weeks in αMEM with 2.5 mM CaCl_2_ in 24-well plates. Cells in the plate were fixed with 2.5% glutaraldehyde and observed with SEM **(A)** and EDS for elemental analysis. **(B)** C K = carbon; O K = oxygen; Ca K = Calcium; P K= Phosphorus; Na K= Sodium. **(C)** EDS spectrum of gating area (yellow box) in B-Grey. Note: Au and Pd from coated gold (Au) and palladium (Pd).

**FIGURE 4:**
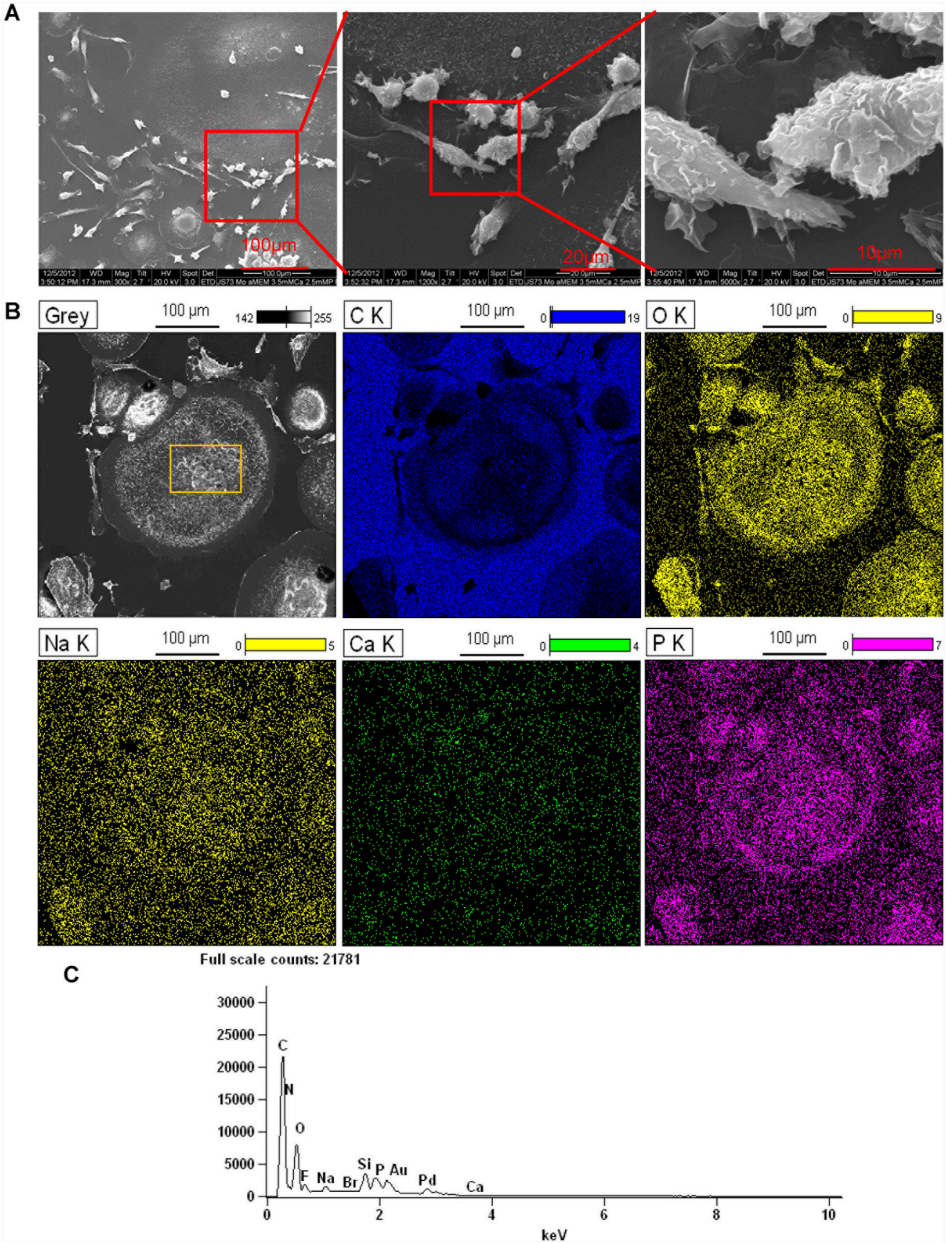
Elemental analysis of osteoclasts in αMEM media with 2.5 mM CaCl_2_. Monocytes were treated with M-CSF/RANKL (both at 25 ng/mL) in 24-well plates in αMEM with 2.5 mM CaCl_2_ for 3 weeks. Cells in the plate were fixed with 2.5% glutaraldehyde and observed with SEM **(A)** and EDS for elemental analysis **(B)**. C K = carbon; O K = oxygen; Ca K = Calcium; P K= Phosphorus; Na K= Sodium. **(C)** EDS spectrum of gating area (yellow box) in B-Grey. Note: Au and Pd from coated gold (Au) and palladium (Pd).

We previously showed that monoosteophils built light refractive, raised nodules on the surface of osteologic discs that stained for inorganic calcium by von Kossa staining after removal of cells (Zhang and Shively, 2010) and phosphorus containing bone nodules with cells on osteologic discs by scanning electron microscopy coupled with energy dispersive microscopy (SEM/EDS) (Zhang and Shively, 2013). We now investigated if monoosteophils can form similar nodules without the requirement for growth on osteologic discs, namely, glass discs coated with hydroxyapatite. Monocytes were cultured in 24-well plates with 5 µM LL-37 for monoosteophil differentiation or with M-CSF/RANKL for osteoclast differentiation in 10% FBS αMEM medium supplemented with 2.5 mM CaCl_2_ for 3 weeks and analyzed by SEM/EDS to investigate the elemental composition of the nodules. In the control group, osteoclasts were used to replace untreated monocytes as control since monocytes not treated with LL-37 continued to undergo cell death, making it difficult to obtain a sufficient number of cells (Zhang and Shively, 2010; Eischen et al., 1991). Our results showed monoosteophil-generated nodules were clearly observed in SEM (Figure 3A). The combination of SEM (fast scanning) with EDS analysis showed that the raised structures consist of oxygen, phosphorus, calcium, and sodium (Figures 3B,C), further verifying the results of ARS and FAM-alendronate staining. In the control group of differentiated osteoclasts under the same conditions, two types of cells (giant cells and small cells) were found with no evidence of nodule formation (Figure 4A). Moreover, EDS analysis did not detect calcium in the osteoclast controls (Figures 4B,C). Thus, monoosteophils generate calcifying/bone-like cells nodules *in vitro* in the absence of osteogenic discs.

### 3.4 Evidence of intracellular nodule formation in monoosteophils

**FIGURE 5:**
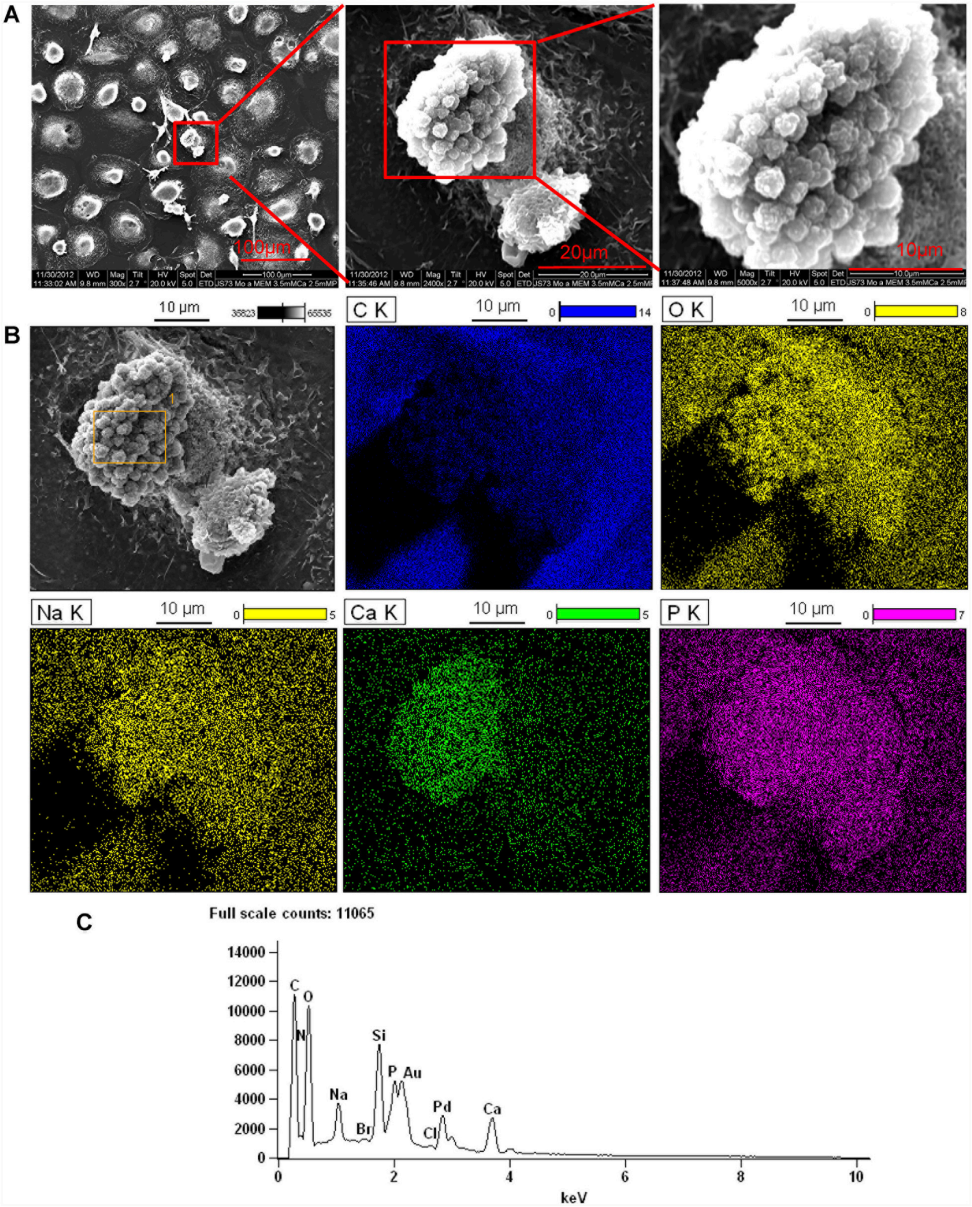
Intracellular nodules formed in monoosteophils consist of phosphorus, calcium, oxygen, and sodium. Monocytes were differentiated to monoosteophils using 5 µM LL-37 in 24-well plate with αMEM and 2.5 mM CaCl_2_ for 3 weeks. Cells in the plate were fixed with 2.5% glutaraldehyde and analyzed with SEM **(A)** and EDS for elemental analysis **(B)**. C K = carbon; O K = oxygen; Ca K = Calcium; P K= Phosphorus; Na K= Sodium. **(C)** EDS spectrum of gating area (yellow box) in B-Grey. Note: Au and Pd from coated gold (Au) and palladium (Pd).

Further studies were performed to determine if the origin of the nodules was intracellular or strictly extracellular. Monocytes treated with LL-37 in 10% FBS αMEM medium supplemented with 2.5 mM CaCl_2_ in 24-well plate for 3 weeks were imaged with SEM (Figure 5A, left panel). In the process of sample preparation, a broken cell was observed in which intracellular granules are clearly seen (Figure 5A, middle panel and right panel). Then, combination of SEM (fast scanning) with EDS were performed to analyze the elemental composition of the intracellular granules. Analysis of the granules by EDS demonstrates they are comprised of oxygen, calcium, phosphorus, and sodium (Figures 5B,C). The results suggest that monoosteophils form bone nodules within the cells.

**FIGURE 6:**
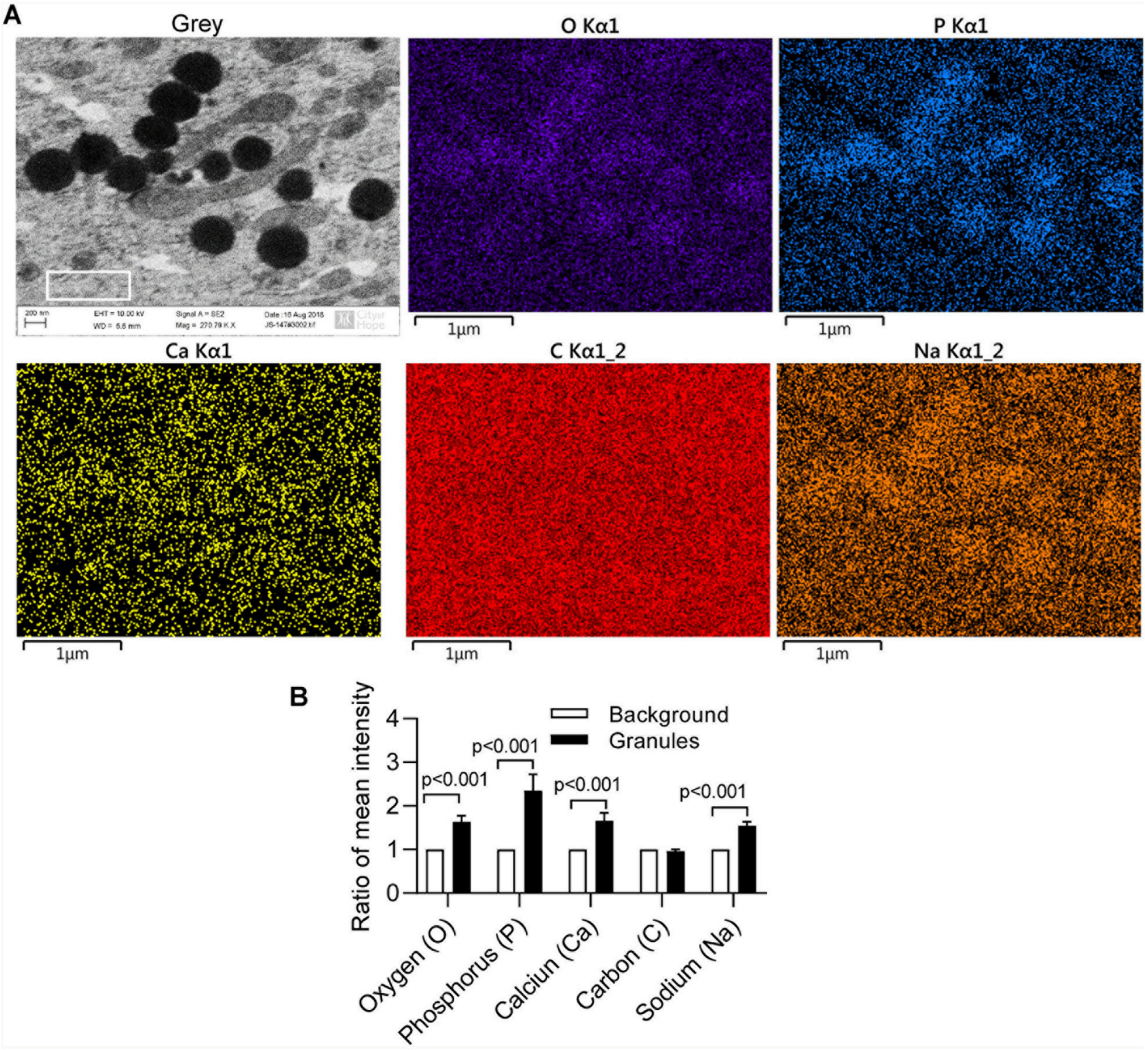
Intracellular nodules formation of monoosteophils in αMEM medium with hydroxyapatite nanoparticles. Monocytes were treated with 5 µM LL-37 in αMEM medium for 1 week and hydroxyapatite nanoparticles were added in the medium for culturing 2 more weeks. Then, cells were harvested, cut to 100 nm thick, and analyzed using SEM and EDS **(A)** with quantification of granule element intensity (**B**, white box gating area of A-Grey in each element as background control) using Image Pro Premier (Media Cybernetics).

In our previous report (Zhang and Shively, 2010; Zhang and Shively, 2013), separated or aggregated granules were shown as spherical to cylindrical shapes with diameters of 0.5–1 µm and lengths of 1–2 µm (Zhang and Shively, 2010) in which EDS analysis showed a composition of phosphorus, calcium, oxygen, and sodium (Zhang and Shively, 2013). When we altered culture conditions by using 10%FBS in αMEM medium with Nano-HA without osteologic discs, monoosteophils were generated from LL-37-treated monocytes in 24-well plate after 3 weeks. Cells were harvested, cut to 100 nm thick, and analyzed by SEM and EDS for the composition of intracellular granules. The granules had spherical shapes with diameters of 0.5–1µm, similar to bone nodules on osteologic disc (Zhang and Shively, 2010). The granules stained dark with uranyl acetate (Figure 6A), while EDS analysis showed granules comprised phosphorus, calcium, oxygen, and sodium (Figure 6A), although the signals of calcium and sodium are weak. Quantification of granule element intensity using Image Pro Premier revealed a significant increase in four elements (Figure 6B). The weak signal is likely due to the limitation of EDS for analyzing intracellular nodules. In the experiment, cells were harvested from plates and processed for TEM specifically to identify unique granules within the cells. The EDS signal is weak because the samples are very thin, not like Figures 3–5, in which the strong EDS signals are derived from multiple granules clustered together outside of cells. Thus, our results suggest that monoosteophils form bone nodules within the cells and then export them extracellularly, in contrast to osteoblast that release ALP to form bone outside cells (Meesuk et al., 2022).

## 4 Discussion

The present study demonstrates that human monoosteophils plated on collagen I sponge scaffolds with hydroxyapatite as a source of calcium and phosphorus have the potential to heal critical-sized calvarial defect in a mouse model. The process of bone healing by monoosteophils occurred within 7 weeks and thereafter switched to a remodeling process (Supplementary Figure 2B). In the *in vivo* study, the newly formed bone tissue appeared healthy, with no signs of neoplasia observed in micro-CT scans and in the harvested bone samples. Cell titration results showed that concentrations of monoosteophils as low as 0.625 × 10^6^ per 19.6 mm^2^ defect still induced bone healing. For the reason of how the low cells count promotes bone tissue formation, first, we know these cells are long lived and continue making new bone *in vitro* for periods out to 6 months, and *in vivo* up to several months. Thus, they do not need to be replenished over time. Second, since new bone formation is inherently a slow process and these cells do not need to be replenished, there may be an upper limit on the number of cells per surface area of template/substrate that can adequately perform their bone forming task. Thus, adding cells to the substrate beyond a certain upper limit may impede their function by competing non-productively for access to the substrate. Third, we know they need both a source of calcium and phosphate, and in the case of a large hole defect, a template to grow on, thus, the number of cells per template area would be expected to be critical. In our previous studies, we showed that monoosteophils accelerated defect healing in a femur drilled-hole mouse model (diameter: 0.9 mm; defect area: 0.636 mm^2^) by using only monoosteopohils (3 × 10^6^ cells) (Zhang and Shively, 2013). In contrast, our current study investigates a much larger defect size of 5 mm (diameter), which is considered a critical-sized defect—that is, a defect too large to heal spontaneously. Notably, we demonstrate that monoosteophils in combination with hydroxyapatite are capable of effectively repairing this critical-sized defect. To summarize both models, we can conclude that: 1) monoosteophils can directly form bone without callus formation (Zhang and Shively, 2013); 2) monoosteophils plus hydroxyapaptite can potentially heal critical-sized defects; 3) sources of calcium and phosphorus in the form of hydroxyapatite are required; and 4) as few as 0.625 × 10^6^ cells can partially heal calviaral bone defects. In the critical-sized defect model, one challenge was the displacement of the collagen sponge from the defect area, likely due to mouse scratching the wound area.

The reason that we choose NOD/SCID mice is that our goal was to implant human monoosteophils into a mouse model that would enable a clinical trial. Since immunocompetent mice would reject human cells, the experiments were performed in immunodeficient mice. Repeating the studies with mouse monoosteophils in immunocompetent mice is an eventual goal to help establish the biological mechanism. However, demonstrating that human cells can perform the same function in the NOD/SCID mice shows the generality of the process and accelerates translation to the clinic. Furthermore, in our study, we found that treating monocytes with LL-37 for just 1 day is sufficient to induce their differentiation into monoosteophils, similar to the effect observed with 6 days of LL-37 treatment *in vitro*. In our previously published study using a drilled hole defect model, we demonstrated that monoosteophils derived from monocytes treated with LL-37 for either one or 6 days produced comparable outcomes in accelerating defect repair (Zhang and Shively, 2010; Zhang and Shively, 2013). Notably, monoosteophils generated after 1 day of LL-37 treatment are only lightly adherent to culture dishes, whereas those treated for 6 days exhibit strong adherence. This difference indicates that monoosteophils induced by a 1-day treatment are much easier to harvest compared to those from a 6-day treatment. Take together, LL-37 promotes the differentiation of monocytes into a novel bone-forming cell type—monoosteophils. These cells contribute to bone repair through intracellular nodule formation, a process distinct from the bone-forming mechanisms of osteoblasts.

Currently, MSCs, osteogenic growth factors and osteoinductive materials are three major approaches used to improve bone repair and regeneration. MSCs are multipotent stromal stem cells that can be harvested from many different sources and differentiated into a variety of cell types, such as preosteogenic chondroblasts and osteoblasts (Oryan et al., 2017). MSC research has made substantial progress in the last three decades since they were first described (Caplan, 1991; Bruder et al., 1994). By June 2020, there were 1,138 registered clinical trials worldwide using MSCs to investigate their therapeutic potential (Rodriguez-Fuentes et al., 2021). The application of bone marrow, placenta, and adipose-derived MSCs in clinical trials around the world were used to treat knee osteoarthritis, but there are also a significant number of clinical trials in the fields of cardiology, pulmonology, and immunomodulation of immune diseases such as host versus graft reaction, rheumatoid arthritis, and Crohn’s disease (Alvaro-Gracia et al., 2017; Panes et al., 2016). Due to the lack of published clinical trials, controversies in the results, and variability in the methods, protocols and treatment strategies, there is still a long way to go in this regard and several issues must be scientifically addressed before it is reasonable to suggest MSCs as a therapeutic option for fracture healing (Oryan et al., 2017).

Circulating CD14^+^ monocytes originate from hematopoietic stem cells in the bone marrow and consist of 5%–10% of circulating white blood cells in humans. The circulating monocytes, also called adult stem cells, are a versatile progenitor cell that gives rise to diverse cell types (Ungefroren et al., 2016). Several cultured human cell populations that originate from circulating monocytes have the capacity to differentiate into nonphagocytic pluripotent stem cell-like cells such as pluripotent stem cells (PSCs) (Zhao et al., 2003), monocyte-derived multipotential cells (MOMC) (Kuwana et al., 2003), CD14^+^CD34^low^KDR^+^ subset (Romagnani et al., 2005), programmable cells of monocytic origin (PCMO) (Ruhnke et al., 2005a; Ruhnke et al., 2005b; Ungefroren and Fandrich, 2010), mesodermal and neuroectodermal lineages (Seta and Kuwana, 2010; Seta and Kuwana, 2007), endothelial progenitor cells (EPC) (Rohde et al., 2006; Kim et al., 2009), smooth muscle-like cells (Albiero et al., 2010), and myeloid calcifying OC^+^BAP^+^ cells (MCCs) (Fadini et al., 2011). Emerging data consistently indicate that circulating calcifying cells are involved in both bone and vascular disease, but several, likely interrelated, phenotypes have been identified (Fadini et al., 2012). Monoosteophils, circulating calcifying cells (Eghbali-Fatourechi et al., 2005; Pirro et al., 2010) and other monocyte-differentiated calcifying cells (Fadini et al., 2011; Fadini et al., 2012) open a new approach to improve bone repair and regeneration. In addition, peripheral blood monocytes have some practical advantages over other types of stem/progenitor cells when they are used for clinical purpose: 1) they can be retrieved from a readily accessible body compartment by a low-invasive procedure; 2) they can be maintained in culture; 3) they have a low risk of tumorigenicity due to their limited proliferative capacity and the lack of *telomerase reverse transcriptase* (hTERT) (Ungefroren et al., 2010); 4) they can be applied to patients in both an autologous and an allogeneic setting, obviating the need for immunosuppression. (Ungefroren et al., 2016).

Although monoosteophils showed positive intracellular staining for osteocalcin, osteonectin, bone sialoprotein II and osteopontin in our previous study, they were negative for bone alkaline phosphatase (ALP) by using both surface and intracellular staining (Zhang and Shively, 2010), which indicates that monoosteophils may use different mechanism to form bone in comparison with the well-known osteoblast. Therefore, we conducted the following *in vitro* experiments to explore the mechanism of bone nodule formation of monoosteophils. After monoosteophils were differentiated and cultured in plate, bone nodule formations were verified by using ARS staining, FAM-alendronate staining and SEM/EDS. Interestingly, some intracellular granules of cells that fractured in the process of preparing SEM samples showed the same components as extracellular bone nodules as previous reported for generation of monoosteophils on osteologic discs (Zhang and Shively, 2010). Furthermore, after comparing extracellular bone nodules on osteologic disc with the intracellular granules of monoosteophils harvested from plastic plates co-cultured with hydroxyapatite nanoparticles (diameter around 63 nm), the sizes of both bone nodules on osteologic disc and intracellular granules of monoosteophils in culturing plate were identical with 0.5–1 µm diameters. Moreover, unique intracellular nodules of monoosteophils were verified as bone nodules by using SEM/EDS. Thus, our results indicated that monoosteophils use an intracellular nodule mechanism to form bone.

The structure of bone arises from a tightly controlled process whereby collagen fibrils secreted by osteoblasts are progressively mineralized by poorly crystalline carbonated apatite (Boonrungsiman et al., 2012). To explain the early bone mineral formation process of osteoblast, various mechanisms have been proposed: 1) a cell-independent process, whereby charged noncollagenous proteins associating with the gap zones in collagen mediate mineral nucleation from ions in solution (Arana-Chavez and Massa, 2004); 2) a cell-controlled mechanism by which vesicles that bud from the plasma membrane accumulate ions extra cellularly mediate calcium phosphate precipitation, and subsequently rupture dispersing their contents on the extracellular matrix (Landis et al., 1977); and 3) an alternative route by which amorphous mineral precursors are transiently produced and deposited within collage fibrils, where they transform into more crystalline apatite platelets (McDonald and Auer, 2006). After decades of support for the former ion-based nucleation models (Boskey, 1998), evidence has recently emerged supporting amorphous mineral precursors in bone mineralization (Boonrungsiman et al., 2012; Mahamid et al., 2008; Mahamid et al., 2010; Nudelman et al., 2010; Dey et al., 2010). Calcium phosphate deposits are known to reside intracellularly in mineralizing cells, notably as granules in mitochondria (Sutfin et al., 1971; Martin and Matthews, 1970) and calcium phosphate-containing vesicles are present in developing mouse bone cells (Boonrungsiman et al., 2012; Mahamid et al., 2011; Iwayama et al., 2019). In comparison with osteoblast-mediated bone apatite (Boonrungsiman et al., 2012), the granules formed by monoosteophils showed unique sizes and shapes when formed in the cytosol.

## 5 Conclusion

In conclusion, our observations highlight the bone repair function and the unique mechanism of bone nodule formation of monoosteophils. These observations may have important implications in facilitating the development of therapeutic applications for clinically challenging bone repair and in understanding pathological mineralization.

## Data Availability

The original contributions presented in the study are included in the article/Supplementary Material, further inquiries can be directed to the corresponding authors.
